# Robust H-Infinity Tracking Control for a Valve-Controlled Hydraulic Motor System with Uncertain Parameters in the Complex Load Environment

**DOI:** 10.3390/s23229092

**Published:** 2023-11-10

**Authors:** Kunwei Lu, Guodong Feng, Beichen Ding

**Affiliations:** 1 School of Intelligent Systems Engineering, Sun Yat-sen University, Shenzhen 510275, China; lukw3@mail2.sysu.edu.cn (K.L.); fenggd6@mail.sysu.edu.cn (G.F.); 2 School of Advanced Manufacturing, Sun Yat-sen University, Shenzhen 510275, China

**Keywords:** valve-controlled hydraulic motor, uncertain parameters, robust h-infinity tracking control

## Abstract

A valve-controlled hydraulic motor system operating in a complex environment is subject to complex load changes. In extreme cases, the load can be regarded as a disturbance signal with complex frequency and strong amplitude fluctuations, which greatly affects the speed stability of the hydraulic motor and reduces the operating efficiency. In this paper, the structure of valve-controlled hydraulic motor systems is analyzed, and a valve-controlled hydraulic motor system model with uncertain parameters is established after considering the actual target parameter error and model linearization error. Different from the common H-infinity control, which regards the load disturbance as external disturbance, this paper presents a robust H-infinity tracking control strategy, which considers uncertain parameters and the load torque of the valve-controlled hydraulic motor system as internal disturbances. The simulation results show that the proposed control scheme has better control characteristics and robustness than the traditional PID control.

## 1. Introduction

Electro-hydraulic servo valve-controlled motor systems are a modern product with the advantages of both electrical servo systems and hydraulic transmission. They are widely used in automobile, marine, metallurgy, petroleum and other industrial scenarios because of their advantages of high-precision control, flexible signal processing, fast response speed and large output power [1]. For example, in the automobile steering system, the valve-controlled hydraulic motor can provide steering power through the hydraulic system, and in the petroleum industry, the valve-controlled hydraulic motor can achieve precise control of the rig, improving the efficiency and safety of the rig operation. However, due to the characteristics of valve-controlled motor systems, such as model non-linearity, parameter uncertainty and load torque interference, the stability, dynamic characteristics and trajectory tracking accuracy of the system will be affected, and it is difficult to achieve high performance control [2].

The speed of a hydraulic motor is an important factor affecting the performance of valve-controlled hydraulic motor systems, but it is difficult to keep the speed of the motor stable in complex operating environments. For example, in the process of rock drill operations, due to complex drilling conditions and large load torque variation range, the hydraulic motor speed is unstable, and when the hydraulic motor speed is lower than the expected speed, the rotary drill can easily become stuck [3]. In the process of polishing barnacles on the bottom of a ship by an underwater hydraulic grinder, it is difficult to maintain a stable speed due to the uneven surface and sharp fluctuation of the load torque, which affects the grinding quality [4,5]. In addition, construction machinery, such as road rollers and road milling machines, will be subject to constant interference from the outside world during operation [6,7].

The common feature of these scenarios is that the load torque changes in a complex manner. In extreme cases, it can be regarded as a disturbance signal with complex frequencies and strong amplitude fluctuations that change with time, which greatly affects the speed stability of the hydraulic motor [8]. Therefore, it is of great value to study the robust control of valve-controlled hydraulic motor systems in complex environments.

Dubonjić [9] studied a PD controller design method based on decomposition, which is controlled by adjusting the proportional and differential parameters of the valve-controlled motor system, but this method does not take into account the non-linear characteristics and disturbance effects of the model. Duan, Hu, Wang et al. [10,11,12] studied auto-disturbance rejection control technology, which can estimate and compensate the disturbances in real time, which improves the disturbance suppression ability of the valve-controlled motor. Yan [13] proposed an end-sliding mode control method, which takes into account the non-linearity of the valve-controlled motor system, and produced a good tracking performance. However, these methods are difficult for solving the problem of high-frequency load disturbance suppression.

The robust H-infinity control is a control strategy based on the optimal control theory [14]. Under the premise of considering the performance and stability of the controller, the robust H-infinity control method reduces the influence of the disturbance on the closed-loop system by limiting the maximum value of the frequency domain gain. Therefore, the robust H-infinity control has filtering characteristics and can suppress the high-frequency components of the load disturbance. This paper uses the H-infinity control method in order to improve the robustness of motor speed when the physical parameters of the valve-controlled motor system are uncertain under complex load disturbances [15,16]. 

In a hydraulic system, the input pressure depends on the output pressure, and the input torque depends on the output torque [17]. Therefore, unlike external additive disturbances, which act directly on the system output and affect the system through the feedback loop, the disturbance of the load torque of the hydraulic motor acts directly on the controlled system itself. The common H-infinity control simply considers the sensitivity function, which essentially regards the load torque as an external additive disturbance, which will result in errors when designing the controller [18,19,20]. Therefore, this paper presents an H-infinity tracking control strategy, which considers the load torque as internal disturbance and uncertain parameters. The simulation data show that the proposed method has good system response characteristics and a good ability to suppress load disturbances in complex environments. In addition, the control strategy proposed in this paper can be repeatedly applied to the system with uncertain parameters and internal disturbances, and it has universal adaptability.

Section 2 introduces the composition of the valve-controlled hydraulic motor system and establishes a system dynamics model with uncertain parameters. In Section 3, this paper proposes a robust H-infinity tracking control method considering uncertain parameters and internal load disturbances. The simulation results are presented in Section 4, and the effectiveness of the proposed method is illustrated through comparison with PID control. The conclusions and discussion are summarized in the last section.

## 2. Valve-Controlled Hydraulic Motor System Model

### 2.1. System Composition

**Figure 1:**
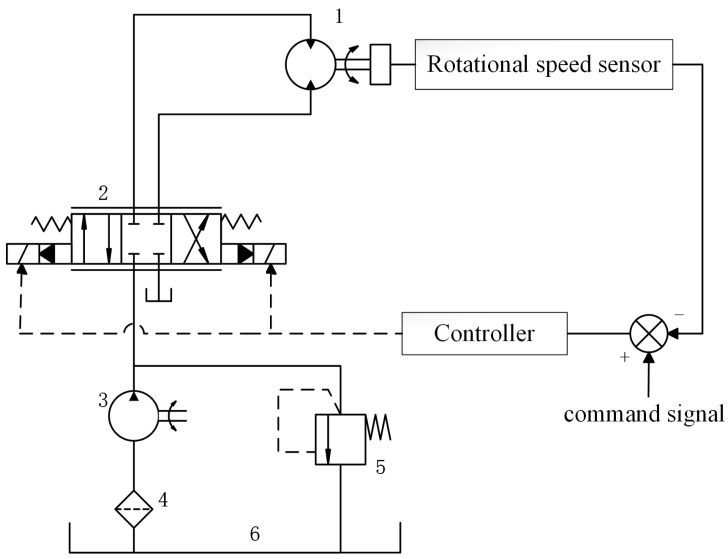
Diagram of valve-controlled hydraulic motor system: 1—hydraulic motor, 2—servo proportional valve, 3—hydraulic pump, 4—filter, 5—overflow valve, 6—fuel tank.

The valve-controlled hydraulic motor system studied in this paper mainly includes a hydraulic motor, servo proportional valve, hydraulic pump, relief valve, tank, speed sensor and controller. The structure is shown in Figure 1.

The hydraulic motor is the executive part of the system, which is responsible for converting hydraulic energy into mechanical energy. The servo proportional valve can control the motor flow by adjusting the opening size; the hydraulic pump is responsible for providing pressure and flow to the system to ensure the normal operation of the system; the relief valve can adjust the output pressure of the pump to ensure the stability of the oil supply pressure of the hydraulic pump; the tank stores the hydraulic oil and acts as an exchanger for the system; the speed sensor is used to measure the speed of the hydraulic motor; and the controller adjusts the opening of the valve according to the feedback signal of speed and pressure to realize speed control.

### 2.2. Dynamic Model

**Figure 2:**
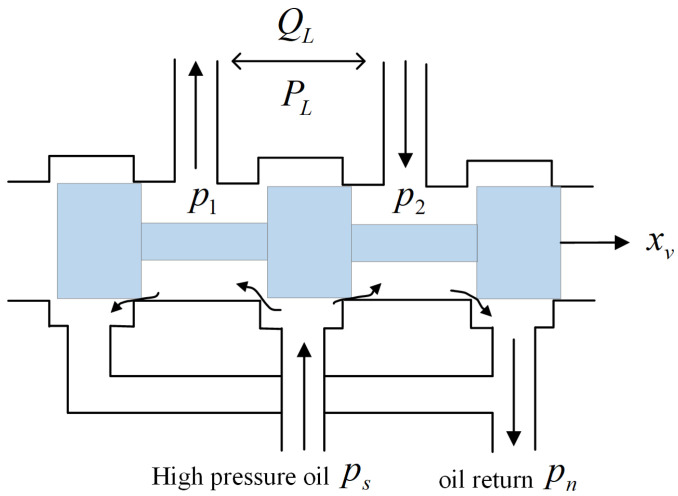
Zero-opening four-way slide valve structure.

First, we consider a zero-opening four-way slide valve structure, as shown in Figure 2 [21]. Assuming that the valve has no internal and external leakage, when the spool is located at the ideal zero position (*x_v_* = 0), each throttle is closed, and the system flow pressure cannot be output.

When the spool moves, the flow equation of the valve can be described by

(1) $$\begin{array}{l} Q_{L}=K_{d}x_{v}\sqrt{p_{s}-p_{L}}(x_{v}>0)\\ Q_{L}=K_{d}x_{v}\sqrt{p_{s}+p_{L}}(x_{v}<0) \end{array}$$

where *P_L_* is the pressure difference between the high-pressure side and low-pressure side; *P_s_* is the supply pressure (assumed to be constant); *K_d_* is the normalized flow coefficient; *Q_L_* is the flow rate of the valve; and *x_v_* is the spool displacement, which is considered a dimensionless variable ranging from −1 to +1.

Considering the unity of the symbols, since the valve is symmetrical, Equation (1) can be rewritten as

(2) $$Q_{L}=K_{d}x_{v}\sqrt{p_{s}-\frac{x_{v}}{\left|x_{v}\right|}p_{L}}$$

Since the control design requires a linear model, it is necessary to differentiate Equation (2), and the linearized flow equation of the valve is given as follows:

(3) $$Q_{L}=K_{q}x_{v}-K_{c}p_{L}$$

where *K_q_* is the flow gain coefficient of the valve, and *K_c_* is the flow pressure gain coefficient of the valve. They can be described by

(4) $$K_{q}=\frac{\partial Q_{L}}{\partial x_{v}}=K_{d}\sqrt{p_{s}-p_{L}}$$

(5) $$K_{c}=-\frac{\partial Q_{L}}{\partial p_{L}}=\frac{K_{d}x_{v}}{2\sqrt{p_{s}-p_{L}}}$$

For a hydraulic fixed gear motor, the volume of the oil inlet cavity and the oil return cavity is approximately equal. When the volume of the hydraulic motor cavity is very small, it can be assumed that the pressure and density at each point in the hydraulic motor are equal and constant. In addition, it is assumed that the oil temperature and bulk elastic modulus are constant, and the related effects of internal friction and oil are not considered, and the pressure loss of the pipeline is ignored.

The flow equation of a fixed displacement hydraulic motor is described as follows [22]:

(6) $$Q_{m}=D_{m}\omega_{m}+C_{m}p_{L}+\frac{V_{t}}{4\beta_{e}}\frac{dp_{L}}{dt}$$

where *V_t_* is the total volume of the pump outlet, motor inlet and pipes; $\beta_{e}$ is the effective bulk modulus of hydraulic oil; *D_m_* is the displacement of the fixed displacement motor; $\omega_{m}$ is the rotational speed of the motor; *C_m_* is the leakage coefficient of the motor; and *Q_m_* is the flow rate of the motor, which is equal to *Q_L_*.

Without considering the elastic load and internal friction force of the hydraulic motor, the total inertia force, viscous resistance and accidental load force of the hydraulic motor and load together constitute the load torque. The dynamic balance equations of the hydraulic motor shaft and load can be described by

(7) $$D_{m}p_{L}=J_{t}\frac{d\omega_{m}}{dt}+B_{m}\omega_{m}+T_{l}$$

where *T_l_* is the load torque; *B_m_* is the damping coefficient of the motor and load; and *J_t_* is the moment of inertia of the motor shaft.

### 2.3. Model with Uncertainties

We define the total discharge coefficient of the valve-controlled motor system *C_t_* = *C_m_* + *K_c_*, state variables *x*_1_ = *P_L_*, *x*_2_ = $\omega_{m}$, control input *u* = *x_v_*, system disturbance input *w_t_* = *T_l_* and intermediate variable *V_b_* = *V_t_*$/4\beta_{e}$. Thus, Equations (3), (6) and (7) can be rewritten as

(8) $$\begin{array}{cc} V_{b}{\dot{x}}_{1} & =-C_{t}x_{1}-D_{m}x_{2}+K_{q}u\\ J_{t}{\dot{x}}_{2} & =D_{m}x_{1}-B_{m}x_{2}-w_{t} \end{array}$$

In the model described in Equation (8), except for the displacement of the fixed displacement motor *D_m_*, which is less affected by the compressibility of the oil and can be assumed to be a fixed value, the other parameters cannot be exactly determined. In the actual system, the total volume *V_t_* is affected by the deformation of the pipeline under different oil pressures, and only theoretical values can be obtained. The total discharge coefficient *C_t_* will vary with the rotation position of the motor. The flow gain *K_q_* is affected by linearization and can only represent the flow gain under a certain working state. The moment of inertia *J_t_* and damping coefficient *B_m_* are determined by the hydraulic motor and load characteristics, but it is difficult to obtain accurate values.

Therefore, the uncertain parameters *V_b_*, *C_t_*, *K_q_*, *J_t_* and *B_m_* are represented in the following form [23]:

(9) $$\begin{array}{cc} V_{b} & ={\bar{V}}_{b}\left(1+p_{v}\delta_{v}\right),C_{t}={\bar{C}}_{t}\left(1+p_{c}\delta_{c}\right),K_{q}={\bar{K}}_{q}\left(1+p_{k}\delta_{k}\right)\\ J_{t} & ={\bar{J}}_{t}\left(1+p_{j}\delta_{j}\right),B_{m}={\bar{B}}_{m}\left(1+p_{b}\delta_{b}\right) \end{array}$$

where ${\bar{V}}_{b},\;{\bar{C}}_{t},\;{\bar{K}}_{q},\;{\bar{J}}_{t}$ and ${\bar{B}}_{m}$ are the nominal values of *V_b_*, *C_t_*, *K_q_*, *J_t_* and *B_m_*, respectively, and *p_v_*, *p_c_*, *p_k_*, *p_j_* and *p_b_* are the maximum uncertain values with $-1\leq \delta_{v},\;\delta_{c},\;\delta_{k},\;\delta_{j},\delta_{b}\leq 1$. In this study, *p_v,c,k,j,b_* = 25%.

They can be represented as the upper linear fraction transformation (LFT) [24]:

(10) $$\begin{array}{cc} V_{b}^{-1} & ={ℱ}_{u}\left(M_{v},\delta_{v}\right),C_{t}={ℱ}_{u}\left(M_{c},\delta_{c}\right),K_{q}={ℱ}_{u}\left(M_{k},\delta_{k}\right)\\ J_{t}^{-1} & ={ℱ}_{u}\left(M_{j},\delta_{j}\right),B_{m}={ℱ}_{u}\left(M_{b},\delta_{b}\right) \end{array}$$

where

(11) $$\begin{array}{l} M_{v}=\left[\begin{array}{ll} -p_{v} & {\bar{V}}_{b}^{-1}\\ -p_{v} & {\bar{V}}_{b}^{-1} \end{array}\right],M_{c}=\left[\begin{array}{cc} 0 & {\bar{C}}_{t}\\ p_{c} & {\bar{C}}_{t} \end{array}\right],M_{k}=\left[\begin{array}{cc} 0 & {\bar{K}}_{q}\\ p_{k} & {\bar{K}}_{q} \end{array}\right]\\ M_{j}=\left[\begin{array}{ll} -p_{j} & {\bar{J}}_{t}^{-1}\\ -p_{j} & {\bar{J}}_{t}^{-1} \end{array}\right],M_{b}=\left[\begin{array}{cc} 0 & {\bar{B}}_{m}\\ p_{b} & {\bar{B}}_{m} \end{array}\right] \end{array}$$

**Figure 3:**
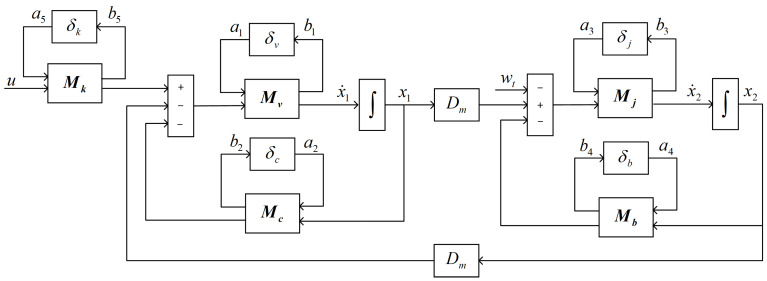
Block diagrams of model with uncertainties.

In order to show the relationship between uncertain parameters, Equation (8) is expressed as a block diagram (Figure 3).

Then, the following equations can be obtained from Figure 3.

(12) $${\dot{x}}_{1}=-\frac{{\bar{C}}_{t}}{{\bar{V}}_{b}}x_{1}-\frac{D_{m}}{{\bar{V}}_{b}}x_{2}-p_{v}a_{1}-\frac{p_{c}}{{\bar{V}}_{b}}a_{2}+\frac{p_{k}}{{\bar{V}}_{b}}a_{5}+\frac{{\bar{K}}_{q}}{{\bar{V}}_{b}}u$$

(13) $${\dot{x}}_{2}=\frac{D_{m}}{{\bar{J}}_{t}}x_{1}-\frac{{\bar{B}}_{m}}{{\bar{J}}_{t}}x_{2}-p_{j}a_{3}-\frac{p_{b}}{{\bar{J}}_{t}}a_{4}-\frac{1}{{\bar{J}}_{t}}w_{t}$$

(14) $$b_{1}={\dot{x}}_{1},b_{2}={\bar{C}}_{t}x_{1},b_{3}={\dot{x}}_{2},b_{4}={\bar{B}}_{m}x_{2},b_{5}={\bar{K}}_{q}u$$

We choose the vector of state variable $x(t)=[x_{1},x_{2}]’$, the vector of output $v(t)=[x_{2}]$, the vector of exogenous inputs $a(t)=[a_{1},a_{2},a_{3},a_{4},a_{5}]’$ and the vector of exogenous outputs $b(t)=[b_{1},b_{2},b_{3},b_{4},b_{5}]’$. The state space model of a valve-controlled hydraulic motor system with uncertain parameters can be obtained in Equations (12)–(14):

(15) $$\begin{array}{l} \dot{x}(t)=Ax(t)+B_{1}a(t)+B_{2}w_{t}(t)+B_{3}u(t)\\ b(t)=C_{1}x(t)+D_{11}a(t)+D_{12}w_{t}(t)+D_{13}u(t)\\ v(t)=C_{2}x(t) \end{array}$$

where

(16) $$\begin{array}{cc} A & =\left[\begin{array}{cc} -{\bar{C}}_{t}{\bar{V}}_{b}^{-1} & -D_{m}{\bar{V}}_{b}^{-1}\\ D_{m}{\bar{J}}_{t}^{-1} & -{\bar{B}}_{m}{\bar{J}}_{t}^{-1} \end{array}\right]\\ \left[\begin{array}{ccc} B_{1} & B_{2} & B_{3} \end{array}\right] & =\left[\begin{array}{ccccccc} -p_{v} & -p_{c}{\bar{V}}_{b}^{-1} & 0 & 0 & p_{k}{\bar{V}}_{b}^{-1} & 0 & {\bar{K}}_{q}{\bar{V}}_{b}^{-1}\\ 0 & 0 & -p_{j} & -p_{b}{\bar{J}}_{t}^{-1} & 0 & -{\bar{J}}_{t}^{-1} & 0 \end{array}\right]\\ {\left[\begin{array}{cc} C_{1} & C_{2} \end{array}\right]}^{\prime } & ={\left[\begin{array}{cccccc} -{\bar{C}}_{t}{\bar{V}}_{b}^{-1} & {\bar{C}}_{t} & D_{m}{\bar{J}}_{t}^{-1} & 0 & 0 & 0\\ -D_{m}{\bar{V}}_{b}^{-1} & 0 & -{\bar{B}}_{m}{\bar{J}}_{t}^{-1} & {\bar{B}}_{m} & 0 & 1 \end{array}\right]}^{\prime }\\ \left[\begin{array}{ccc} D_{11} & D_{12} & D_{13} \end{array}\right] & =\left[\begin{array}{ccccccc} -p_{v} & -p_{c}{\bar{V}}_{b}^{-1} & 0 & 0 & p_{k}{\bar{V}}_{b}^{-1} & 0 & {\bar{K}}_{q}{\bar{V}}_{b}^{-1}\\ 0 & 0 & 0 & 0 & 0 & 0 & 0\\ 0 & 0 & -p_{j} & -p_{b}{\bar{J}}_{t}^{-1} & 0 & -{\bar{J}}_{t}^{-1} & 0\\ 0 & 0 & 0 & 0 & 0 & 0 & 0\\ 0 & 0 & 0 & 0 & 0 & 0 & {\bar{K}}_{q} \end{array}\right] \end{array}$$

## 3. H-Infinity Tracking Control

### 3.1. H-Infinity Control Method with Uncertainty

Different from traditional PID control, which does not require accurate identification of the model and is controlled purely according to the error values, H-infinity control needs to solve the minimal infinite norm of the transfer function from the external input parameters to the controlled output according to the system model, which has the characteristics of optimal control and strong robustness. In the common H-infinity mixed sensitivity method, the disturbance often exists as an additive disturbance outside the controlled object, but for the valve-controlled hydraulic motor system, the load disturbance acts directly on the system. If it is regarded as an external disturbance, load observation is required, but the load observation method essentially introduces an estimated load with uncertain factors, which will lead to additional errors in the system [25]. However, when the load disturbance is regarded as internal disturbance as part of the system, no additional error will be generated, which can effectively improve the robustness of the system. The following section introduces a design method of an H-infinity tracking controller when the load disturbance is considered an internal disturbance.

**Figure 4:**
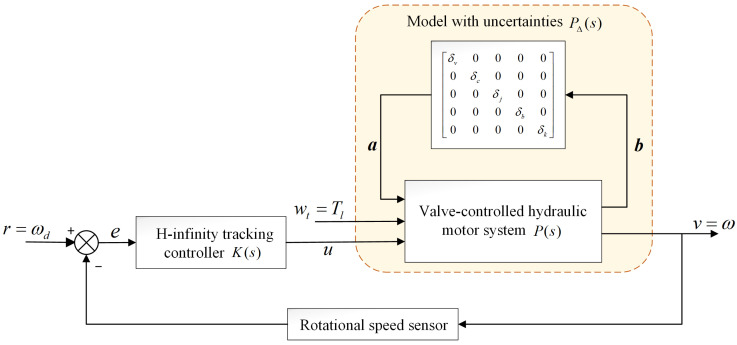
Framework of valve-controlled hydraulic H-infinity tracking control.

The overall control framework of the valve-controlled hydraulic motor H-infinity tracking control used in this research is presented in Figure 4. As shown in Figure 4, $P_{∆}(s)$ is the upper linear fractional transformation structural model of the nominal system P(s) and the uncertainty ∆, whose inputs are load torque *T_l_* and control input *u*, and the output is motor rotation speed *v.*

The input/output relationship of P(s) can be described by

(17) $$\left[\begin{array}{c} b\\ v \end{array}\right]=\left[\begin{array}{ccc} p_{11} & p_{12} & p_{13}\\ p_{21} & p_{22} & p_{23} \end{array}\right]\left[\begin{array}{c} a\\ w_{t}\\ u \end{array}\right]$$

According to Equation (15)

(18) $$p_{ij}=C_{i}{\left(sI-A\right)}^{-1}B_{j}+D_{ij}\;(i=1,2;j=1,2,3)$$

We define a coefficient matrix of uncertainties as

(19) $$\Delta =\mathrm{diag}\left(\left[\delta_{v},\delta_{c},\delta_{j},\delta_{b},\delta_{k}\right]\right)$$

∆ represents structural uncertainty and satisfies the norm condition ${\left\|∆\right\|}_{\infty }\leq 1$ [26]. For this work, we consider δ($\delta_{v}=\delta_{c}=\delta_{k}=\delta_{j}=\delta_{b})$. Equation (17) can be described by

(20) $$\begin{array}{l} b=p_{11}a+p_{12}w_{t}+p_{13}u\\ v=p_{21}a+p_{22}w_{t}+p_{23}u \end{array}$$

It is obvious that a=∆b; by eliminating a and b in Equation (19), the system output v can be rewritten as

(21) $$v=p_{\Delta 1}w_{t}+p_{\Delta 2}u$$

where

(22) $$\begin{array}{l} p_{\Delta 1}=p_{21}\Delta {\left(I-p_{11}\Delta \right)}^{-1}p_{12}+p_{22}\\ p_{\Delta 2}=p_{21}\Delta {\left(I-p_{11}\Delta \right)}^{-1}p_{13}+p_{23} \end{array}$$

**Figure 5:**
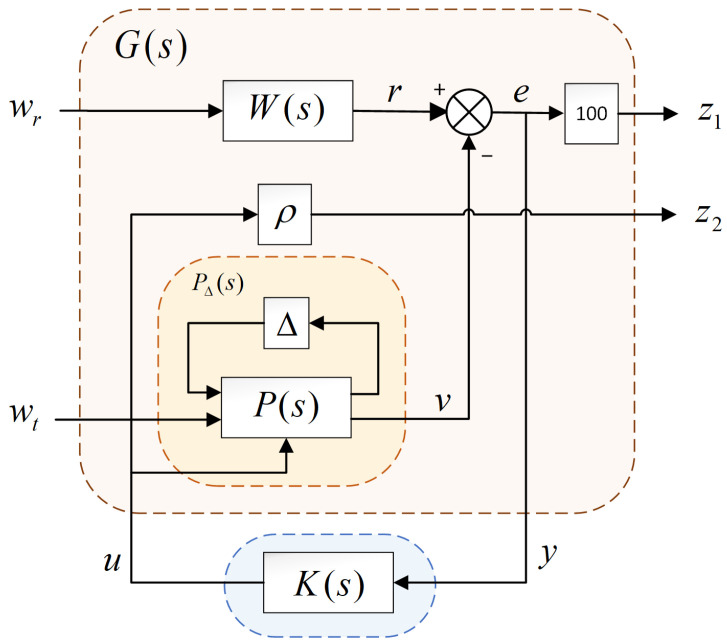
Typical H-infinity control configuration of system.

The H-infinity control method finds the controller *K*(s), which makes the load torque disturbance have the least influence on the system output under the premise of considering the stability and tracking performance. According to a typical H-infinity control theory, the structure shown in Figure 4 is transformed to the structure shown in Figure 5 [27].

As shown in Figure 5, the actual controlled object $P_{∆}(s)$ is the hydraulic system to be controlled; $w_{r}$ is the actual input and target tracking trajectory; $w_{t}$ is the load disturbance input of the hydraulic system; y is the actual input of the controller K(s); $z_{1}$ is the controlled output; and $z_{2}=\rho u$ is the energy penalty term of the regular controller, which makes the system exist. W(s) ensures the penalty weight of the limited energy of the reference input ***r***, so that the designed controller K(s) can have a waveform integer effect on the actual input $w_{r}$.

Using the H-infinity tracking controller K(s), the infinite norm of the closed-loop transfer function from load disturbance $w_{t}$ to system error $z_{1}$ can be minimized, so that the system has the ability to resist load disturbances. Meanwhile, in order to ensure that the output error signal is small enough under the influence of disturbances, a weight factor of 100 is added after the error signal.

In Figure 5, it is not difficult to show that $z_{1}=100(r-v)$, $z_{2}=\rho u$, and y=r−v; combined with Equation (21), the input/output relationship of the generalized controlled plant G(s) and the controller equation can be expressed as follows:

(23) $$\left[\begin{array}{c} z_{1}\\ z_{2}\\ y \end{array}\right]=\left[\begin{array}{c} 100(r-v)\\ \rho u\\ r-v \end{array}\right]=\left[\begin{array}{ccc} 100W & -100p_{\Delta 1} & -100p_{\Delta 2}\\ 0 & 0 & \rho \\ W & -p_{\Delta 1} & -p_{\Delta 2} \end{array}\right]\left[\begin{array}{c} w_{r}\\ w_{t}\\ u \end{array}\right]=G\left[\begin{array}{c} w_{r}\\ w_{t}\\ u \end{array}\right]$$

(24) u=Ky

where

(25) $$\begin{array}{cc} G_{11} & =\left[\begin{array}{cc} 100W & -100p_{\Delta 1}\\ 0 & 0 \end{array}\right],G_{12}=\left[\begin{array}{c} -100p_{\Delta 2}\\ \rho \end{array}\right]\\ G_{21} & =\left[\begin{array}{cc} W & -p_{\Delta 1} \end{array}\right],G_{22}=\left[-p_{\Delta 2}\right] \end{array}$$

The closed-loop transfer function from vector $w=[w_{r},w_{t}]’$ to vector $z=[z_{1},z_{2}]’$ is given by

(26) $$T_{zw}(s)=G_{11}+G_{12}K{\left(I-G_{22}K\right)}^{-1}G_{21}=F_{l}(G,K)$$

where $F_{l}(G,K)$ is the lower linear fractional transformation (LFT) [24] of ***G*** and ***K***.

Therefore, the problem of H-infinity tracking control for a valve-controlled hydraulic motor system with uncertain parameters can be attributed to the design problem of typical H-infinity structures corresponding to the generalized controlled object G(s). K(s) can be obtained by solving the following optimal H-infinity problem [28].

(27) $$\underset{K\;\mathrm{stabilising}\;}{\min}{\left\|F_{l}(G,K)\right\|}_{\infty }$$

### 3.2. Design of H-Infinity Tracking Controller

We selected MOOG G7613005B [29] and STANLEY GR29 [30] as the models for the servo proportional valve and the fixed displacement motor, respectively. The physical parameters of the valve-controlled hydraulic motor system are listed in Table 1. The motor displacement $D_{m}$, the total volume of hydraulic system ${\bar{V}}_{t}$, the moment of inertia ${\bar{J}}_{t}$ and the oil supply pressure $P_{s}$ are calculated from the product specifications. The effective bulk modulus $\beta_{e}$ and the damping coefficient ${\bar{B}}_{m}$ are empirical values. The total discharge coefficient ${\bar{C}}_{t}$ is the sum of the calculated flow pressure gain coefficient, and the motor leakage coefficient is given by the product specification. The flow coefficient ${\bar{K}}_{q}$ is calculated through the combination of linearized flow equation and normalized spool moving distance.

As shown in Figure 5, the weighting matrix W(s) is a stable real rational function, which can ensure that the reference input energy is limited and has the characteristics of low-pass filtering. When there is no weight factor ρ in the system, the controller obtained is an irregular controller, and the amplitude of the control signal is infinite, which cannot be realized. Therefore, the weight factor ρ makes the H-infinity method have a solution, and the control quantity can be limited to prevent serious saturation phenomena in the control process [31]. Based on the experimental method, the W(s) and ρ can be obtained as

(28) $$W(s)=\frac{1}{s+100},\rho =0.001$$

The H-infinity controller is designed for one of the working conditions of the valve-controlled hydraulic motor system, and the uncertainty applied to the parameters has a magnitude of 10%. Through the hinfsyn function in the MATLAB robust control toolbox, the state space form of H-infinity controller K(s) can be obtained:

(29) $$K_{A}=\left[\begin{array}{ccc} -4.43\times 10^{4} & -4.1\times 10^{4} & 3.67\times 10^{8}\\ -2.43\times 10^{4} & -2.25\times 10^{4} & 2.01\times 10^{8}\\ -620 & -305 & -1.06\times 10^{6} \end{array}\right]$$

(30) $$K_{B}=\left[\begin{array}{c} -22.17\\ 44.35\\ 6.15\times 10^{3} \end{array}\right]$$

(31) $$K_{C}=\left[\begin{array}{ccc} -49.33 & -46.11 & 4.13\times 10^{5} \end{array}\right]$$

(32) $$K_{D}=0$$

## 4. Simulation Results

When the valve-controlled hydraulic motor tool works in a complex environment, the load condition can be divided into an abrupt load and AC load, and, in extreme cases, it can be regarded as a signal with time-varying complex frequency and strong amplitude fluctuation [32]. In order to evaluate the performance of the proposed control scheme, the conditions of no-load torque, load torque mutation, high-amplitude single-frequency load torque and high-amplitude complex-frequency load torque were considered, and the step response dynamic analysis was carried out under the condition of 100 rad/s as the target speed. At the same time, PID controllers with a proportional coefficient, integral coefficient and differential coefficient of 10, 0.1 and 0.05 were selected for comparison.

### 4.1. Without Load Torque

**Figure 6:**
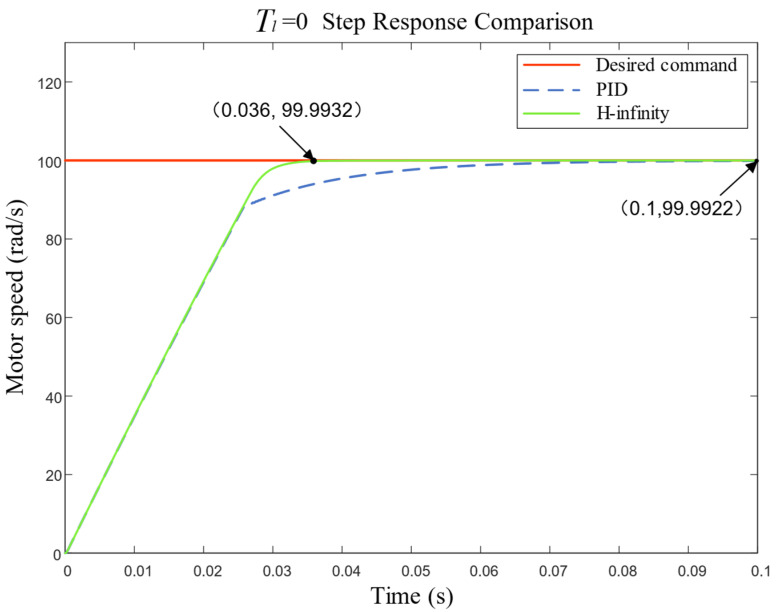
Comparison of motor speed step response without load torque.

When the load torque $T_{l}=0$, the step response of the motor speed under the two controllers is shown in Figure 6, and the specific rise time and steady-state error are shown in Table 2.

In Table 2, the step response of both controllers did not produce overshoot, and both could meet the requirements of system speed tracking. The step response rise time of the motor speed of the H-infinity controller was 0.036 s, which is obviously smaller than the rise time of 0.1 s under the PID controller, and the system response time was significantly improved. However, the steady-state error of the H-infinity controller was 0.0068 rad/s, which was not much different from that of the PID controller (0.0078 rad/s).

### 4.2. Load Torque Abrupt Change

In the actual work, the maximum torque, which the hydraulic motor can output, has an upper limit, so the load torque it can withstand also has an upper limit. When the motor is rotating at a constant speed, Equation (7) can be rewritten as

(33) $$T_{g}=D_{m}p_{L}=B_{m}\omega_{m}+T_{l}$$

where *T_g_* is the motor output torque. 

The upper limit of the load pressure *P_L_* depends on the system oil supply *P_S_*; when the motor speed is 100 rad/s, the maximum load of the hydraulic motor is given by

(34) $$T_{l\max}=D_{m}p_{s}-B_{m}\omega_{m}=34.48N\cdot m$$

Considering the actual working conditions, the maximum load torque was set to 30 N·m during simulation.

**Figure 7:**
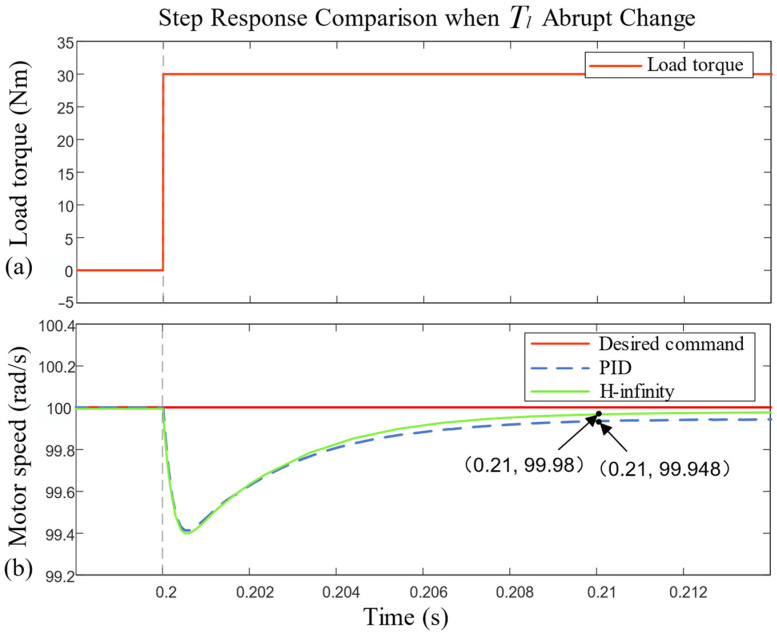
Comparison of motor speed step response when load torque abruptly changes.

As can be seen in Figure 7, when the load torque $T_{l}$ changes from 0 N·m to 30 N·m at 0.2 s and is maintained, both controllers quickly produce a fluctuation with a 0.6 rad/s amplitude and quickly return to the steady state. The time required for steady-state restoration and the final steady-state error are shown in Table 3.

In Table 3, the time required for the H-infinity controller to restore a steady state is basically the same as that for the PID controller, but it has a smaller final steady-state error. The H-infinity controller has better resistance to a load disturbance.

### 4.3. Single-Frequency Load Torque Condition

**Figure 8:**
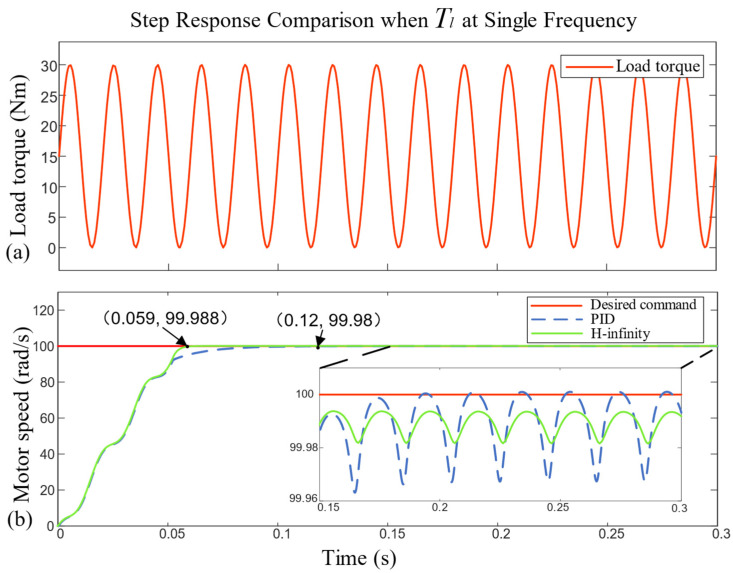
Comparison of motor speed step response under single-frequency load torque.

As shown in Figure 8, when the load torque $T_{l}=15\sin\left(100\pi t\right)+15$, enlarging the speed curve between 0.15 s and 0.3 s, the speed curve of both controllers carried a sinusoidal signal with a frequency of 50 Hz. The step response rise time, peak-to-peak value of the sinusoidal signal and average steady-state error are shown in Table 4.

In Table 4, the rise time of the H-infinity controller was 0.059 s, which was obviously better than the 0.12 s under the traditional PID controller. In addition, the peak and average steady-state error of the sinusoidal signal of the H-infinity controller were smaller than those of the PID controller, which could better restrain a torque disturbance of a single-frequency load.

### 4.4. Complex-Frequency Load Torque Condition

**Figure 9:**
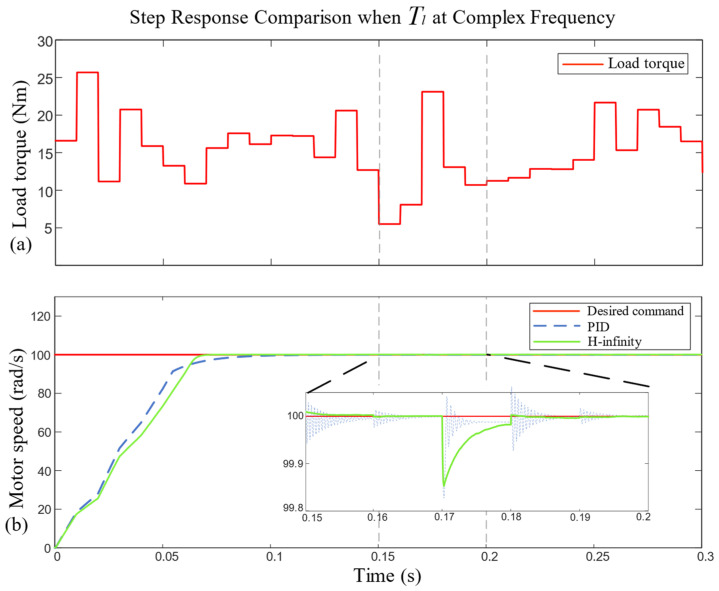
Comparison of step response of motor speed under load torque with complex frequency.

In the actual operation, the load torque $T_{l}$ can be regarded as a signal, which varies with time, has a complex frequency and strong amplitude fluctuations. Therefore, the load torque $T_{l}$ was assumed to be band-limited white noise; the noise power was set to 0.2, the sampling time to 0.01 s, with a bias of 15 N·m; and the load torque $T_{l}$ ranged from 0 N·m to 30 N·m. The load torque curve and hydraulic motor speed step response curve are shown in Figure 9.

As can be seen in Figure 9, when the load torque $T_{l}$ was band-limited white noise, the speed curve of the PID controller oscillated when $T_{l}$ suddenly changed, and there was a maximum oscillation amplitude of 0.18 rad/s error, which was caused by the sensitivity of the PID controller to the direction of the sudden change in load torque. Even if the PID proportional gain is reduced, it cannot be eliminated, which greatly affects the control stability of the system.

Although the maximum fluctuation amplitude of the H-infinity controller was 0.17 rad/s, it was basically the same as that of the PID controller, but there was no oscillation phenomenon. The speed curve of the H-infinity controller pulsed when the load torque changed, and it then quickly returned to the steady state, with a good tracking effect. However, when the load torque changed dramatically in a short and continuous period, the H-infinity controller extended the reaction time and returned to the steady state at about 0.01 s. This reaction time depends on the size of the weight factor before $z_{1}$ in Figure 5. The larger the weight factor, the shorter the reaction time.

Given the above simulation results, it can be concluded that under different load torque disturbance conditions, the proposed robust H-infinity tracking control scheme with uncertain parameters has good rejection and tracking performance.

## 5. Discussion and Conclusions

In this paper, the structure of a valve-controlled hydraulic motor system was analyzed in detail, and the system model with uncertain parameters was established considering the actual object parameter error and model linearization error. Due to the complex applications of valve-controlled hydraulic motor tools, such as rock drilling, cleaning seabed barnacles, etc., the load torque can be regarded as an internal disturbance signal with a complex frequency and a high amplitude. Therefore, the internal disturbance robust H-infinity tracking control strategy with uncertain parameters was proposed, and the corresponding controller was solved.

The analysis of the simulation data revealed that the robust H-infinity tracking control scheme with uncertain parameters could make the motor speed accurately follow the given expected speed and had strong robustness to uncertain parameters of and load torque disturbances to the valve-controlled motor system. Due to the limitation of the experimental environment, kinetic equations were used for modeling, without more detailed modeling, and physical experimental verification has yet to be completed, making the completeness of the discussion in this paper somewhat regrettable. 

## Figures and Tables

**Table 1:** Parameter values of the hydraulic models.

Parameter	Symbol	Value
Motor displacement	$D_{m}$	2.28 × 10^−6^ (m^3^/rad)
Total volume of hydraulic system	${\bar{V}}_{t}$	1.1 × 10^−4^ (m^3^)
Effective bulk modulus	$\beta_{e}$	0.9 × 10^9^ (N/m^2^)
Total discharge coefficient	${\bar{C}}_{t}$	4 × 10^−12^ (m^5^/N/s)
Flow gain coefficient	${\bar{K}}_{q}$	3.5 × 10^−7^ (m^3^/s)
Moment of inertia	${\bar{J}}_{t}$	0.01 (kg·m^3^)
Damping coefficient	${\bar{B}}_{m}$	0.02 (N·s·m)
Fuel supply pressure	$P_{s}$	1.6 × 10^7^ (Pa)

**Table 2:** Performance index of motor speed step response without load torque.

Controller	Rise Time (s)	Steady-State Error (rad/s)
PID	0.1	0.0078
H-infinity	0.036	0.0068

**Table 3:** Performance index of motor speed step response under abrupt change in load torque.

Controller	Time to Steady-State Recovery (s)	Steady-State Error (rad/s)
PID	0.01	0.052
H-infinity	0.01	0.02

**Table 4:** Performance index of motor speed step response under single-frequency load torque.

Controller	Rise Time (s)	Peak-To-Peak Value (rad/s)	Average Steady-State Error (rad/s)
PID	0.12	0.035	0.02
H-infinity	0.059	0.012	0.012

## Data Availability

The data presented in this study are available on request from the corresponding author.
